# “When the Bug Cannot Be Killed”—The Rising Challenge of Antimicrobial Resistance

**DOI:** 10.3390/medicines4020040

**Published:** 2017-06-14

**Authors:** Richard Ofori-Asenso

**Affiliations:** Research Unit, Health Policy Consult, P.O. Box WJ 537, Weija-Accra, Ghana; asensox215@gmail.com; Tel.: +233-546-228-756

**Keywords:** antimicrobial resistance, superbugs, international health

## Abstract

Antimicrobial resistance is a major global health issue that has the potential to reverse the substantial progress made against infectious diseases over the past several decades. We need strategic measures to deal with this challenge, including an intensification of public funding for research into anti-microbial agents and their alternatives, stricter mechanisms to minimize antimicrobial misuse within both clinical and non-clinical settings, and support for the development of country-level initiatives. Only with sustained, concerted, and coordinated global efforts are we likely to overcome the current and future challenges posed by these emerging “superbugs”.

Dear Editor,

On 13 January 2017, the Centers for Disease Control and Prevention (CDC) reported that a woman in Nevada had died from an infection (contracted while in India) resistant to all available antibiotics in the United States [1]. While this deservedly attracted significant international attention, the emergence of “superbugs” resistant to available antibiotics are increasingly being reported by researchers across the world. In 2016, a 56-year-old man from rural Victoria in Australia with no history of hospital contact or international travel was reported to have died from *Klebsiella pneumoniae* infection that was resistant to all antibiotics in the country [2]. In February 2017, Wang and colleagues also reported the emergence of a “superbug” *Escherichia coli* resistant to last resort antibiotics in China [3].

These reports highlight the scale of the antimicrobial resistance crisis that is confronting the world. A number of antibiotics once regarded as “wonder drugs” are no longer working, and for many others, there is an uncertain future of their potency. As indicated by the World Health Organization (WHO), “a post-antibiotic era—in which common infections and minor injuries can kill—far from being an apocalyptic fantasy” is becoming a “real possibility” in our time [4]. A recent report from a review committee commissioned by the British government estimates that, by 2050, nearly 10 million people are likely to die from drug-resistant infections if the current trends persist and if pragmatic measures are not urgently implemented [5]. The economic loss of such an occurrence has been estimated to be around US$100 trillion globally [5].

Yet, global efforts towards addressing the antimicrobial resistance crisis have been generally slow and inadequate [6]. Ultimately, we can only slow but not entirely stop antibiotic resistance; to achieve this, over time, newer and more powerful antibiotics will be required. However, the traditional reliance on pharmaceutical companies to bring out much needed newer and highly potent antibiotics that can tackle resistant pathogens is no longer fit for purpose. Indeed, over the past two decades, many large pharmaceutical companies have abandoned or limited their involvement in the antibiotic field. Such shifts are a result of a myriad of factors, including scientific and regulatory challenges, but are largely driven by economic considerations, as investments in development of therapies for chronic ailments such as diabetes are considered to offer better returns [7,8]. Recent analysis by Leonard et al. [9] suggests that there may be some glimpse of hope, as nearly 30 new antibiotic indications are in development and hold the prospect of making it into the market in the next five years. Nonetheless, these are not necessarily new classes of antibiotics but derivatives of existing ones, and many are not active against high-risk systemic, urinary tract, and respiratory infections [9]. 

To effectively address the discrepancies in antibiotic research and development as well as public health needs, there is an urgent requirement for increased public funding and promotion of integrated collaboration between industry, government, academia, and non-profit players. This will be essential to build on the already extensive resources available. According to Singh [10], there is genetically validated data on nearly 350 antibacterial targets. However, less than 20 of such targets are the focus of currently marketed antibiotics. There is therefore great opportunity for the discovery and development of new molecules with novel mechanisms of action devoid of cross-resistance [10]. New public–private initiatives, such as the Combating Antibiotic-Resistant Bacteria Accelerator (CARB-X) [11], which aims to provide funding and support to scientists and researchers to facilitate antibacterial product development, offer some hope towards securing a sustainable pipeline of antibiotics. To bring some focus in antibiotic research and development, the WHO has also recently published a list of antibiotic-resistant “priority” pathogens, highlighting areas that require urgent attention [12].

However, even with intensified efforts, it is likely that achieving a stable pipeline and reliable supply of antibiotics into the market will take many years. Beyond this, a wider approach is needed, including the intensification of efforts to minimize infection with, spread of, and selection of resistant pathogens. Some evidence suggest that areas such as surveillance still attract less funding with most resources being channeled towards the area of therapeutics [13]. There is therefore the need for a more balanced approach to ensure that all areas with the potential to contribute to dealing with issues of antimicrobial resistance are adequately resourced. 

Measures to improve the rational use of antimicrobial agents need to be intensified both within clinical settings and in other areas such as farming [14]. Such measures will be essential to prolonging the lifespan of available antibiotics, especially since the time between the introduction of an antibiotic and the development of resistance has become increasingly shorter since 1970 [15]. The WHO has developed a global action plan to tackle antimicrobial resistance, which outlines different approaches including optimal antimicrobial utilization and countries are expected to develop national plans [16]. However, many countries (particularly in developing regions such as Sub-Saharan Africa) [17] still lack such national plans, and it will be essential to intensify support to such countries so as to enable the development of local measures that meet country-specific needs and circumstances. Clearly, the declines in antibiotic development along with the challenges of their use necessitate the need for consideration of alternative sources of infection control beyond antibiotics both in human clinical settings and in agriculture [18]. For instance, increased research and funding for the development of vaccines and probiotics may be necessary.

Nearly nine decades after the discovery of penicillin by the Scottish pharmacologist Sir Alexander Fleming [19], we are at the forefront of a new battle faced with dangerous “superbugs” that are impervious to our best antibiotics. The rising antimicrobial resistance not only is a threat to the health of the global population, but also has the potential to reverse the substantial progress made against infectious diseases. We need strategic measures to address the challenge including the intensification of public funding for research, the development of mechanisms to minimize antimicrobial misuse within both clinical and non-clinical settings, and support for the development of country-level initiatives. Innovative measures for infection control beyond the use of antibiotics are additionally required. As the Nobel Laureate Joshua Lederberg once wrote, “the future of humanity and microbes will evolve as episodes...of our wits versus their genes” [20]. Thus, it is only with sustained, concerted, and coordinated global efforts are we likely to overcome the current and future challenges posed by these emerging “superbugs”.
